# Molecular mechanisms in DM1 — a focus on foci

**DOI:** 10.1093/nar/gkv029

**Published:** 2015-01-20

**Authors:** Olof Joakim Pettersson, Lars Aagaard, Thomas Gryesten Jensen, Christian Kroun Damgaard

**Affiliations:** 1 Department of Biomedicine, Aarhus University, Wilhelm Meyers Allé 4, Building 1240, DK-8000 Aarhus C, Denmark; 2 Department of Molecular Biology and Genetics, Aarhus University, C.F. Møllers Allé 3, Building 1130, DK-8000 Aarhus C, Denmark

## Abstract

Myotonic dystrophy type 1 is caused by abnormal expansion of a CTG-trinucleotide repeat in the gene encoding Dystrophia Myotonica Protein Kinase (DMPK), which in turn leads to global deregulation of gene expression in affected individuals. The transcribed mRNA contains a massive CUG-expansion in the 3′ untranslated region (3′UTR) facilitating nucleation of several regulatory RNA-binding proteins, which are thus unable to perform their normal cellular function. These CUG-expanded mRNA–protein aggregates form distinct, primarily nuclear foci. In differentiated muscle cells, most of the CUG-expanded RNA remains in the nuclear compartment, while in dividing cells such as fibroblasts a considerable fraction of the mutant RNA reaches the cytoplasm, consistent with findings that both nuclear and cytoplasmic events are mis-regulated in DM1. Recent evidence suggests that the nuclear aggregates, or ribonuclear foci, are more dynamic than previously anticipated and regulated by several proteins, including RNA helicases. In this review, we focus on the homeostasis of DMPK mRNA foci and discuss how their dynamic regulation may affect disease-causing mechanisms in DM1.

## INTRODUCTION

Myotonic dystrophy type 1 (DM1) is a multisystemic disease and the most common of the muscular dystrophies among adults. The symptoms and clinical findings of this dominantly inherited disease include myotonia (hyperexcitability of skeletal muscle), muscle wasting, cardiac conduction defects, cataracts and insulin resistance, recently reviewed in (1–4). The disease is caused by a trinucleotide expansion (CTG) in the gene encoding Dystrophia Myotonica Protein Kinase (DMPK) (5–9). Cognitive dysfunction and mental retardation has also been documented in the most severe cases of DM1, which is reminiscent of the congential form of DM1 (cDM1). The *DMPK* gene of healthy individuals contains between 5 and 38 CTG repeats, while those harboring between 39 and 50 repeats are considered premutation alleles (3). Clinically affected individuals have *DMPK* genes harboring between 50 and several thousands of repeats among the most severely affected. In line with this, disease severity increases while age of onset decreases with an increasing number of repeats (1,3,10). The CTG expansion lies within a region coding for the 3′ untranslated region (3′UTR) of the DMPK mRNA that is thought to adopt an elongated metastable stem-loop structure, which is likely dynamically regulated by a multitude of RNA-binding proteins *in vivo* (11–15). Although DM1 is among the relatively well-characterized muscular dystrophies, many questions regarding the molecular disease-causing mechanisms remain unanswered. Early experiments strongly suggested a ‘gain-of-function’ mechanism, since expression of CUG-expansions is not only necessary but also sufficient to drive and recapitulate a severe myotonic dystrophy phenotype in transgenic mice (16). In line with this, earlier studies of knockout mice for DMPK (or its flanking genes *SIX5* and *DMAHP*) demonstrated that DMPK haploinsufficiency could not account for the disease severity inflicted by CUG-expansions (17–20).

**Figure 1.**
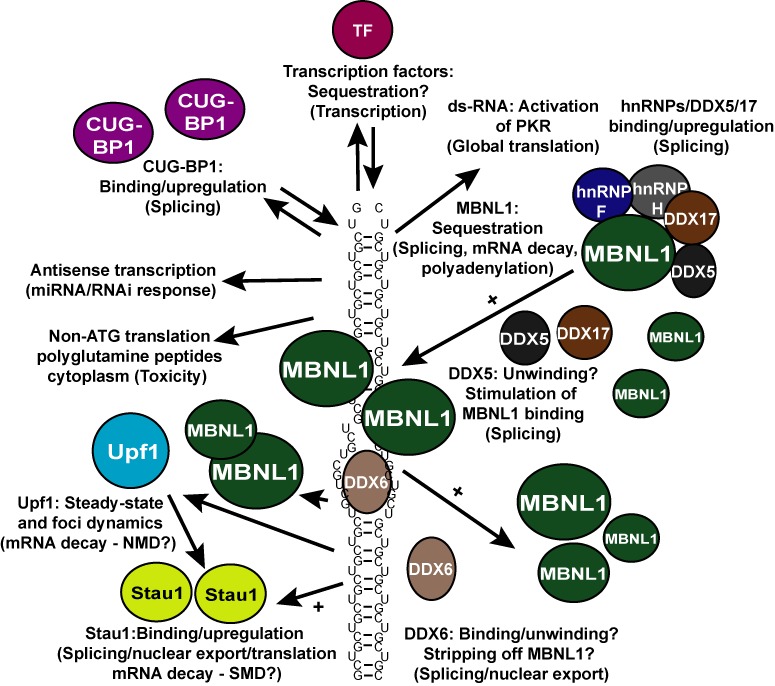
Proposed disease-related mechanisms of CUG-expansions in DM1. Massive CUG-expansions in the 3′UTR of DMPK mRNA likely fold into a metastable hairpin structure (simplified here as a small number of CUG-repeats), which facilitates binding/sequestration of several factors including those depicted by colored circles/ovals. Examples of factors and the processes they affect (in parentheses) are presented. One important effect of this factor binding/sequestration is a misregulation of several specific splicing events causing adverse multisystemic phenotypes in affected individuals. TF = transcription factor. Mainly MBNL1 (green oval) is sequestered by CUG-hairpins, enhanced by the action of one or two of its interacting proteins, DEAD-box helicases DDX5/DDX17, which in turn promotes foci formation. DDX6 transiently interacts, unwinds the hairpin and potentially releases bound MBNL1 to allow for increased nuclear export and translation, which is also stimulated by Staufen 1 (Stau1). These proteins therefore likely counteract the function of DDX5. See text for references and further details.

How does expression of these seemingly simple CUG-expansions in the 3′ untranslated region of an mRNA create such adverse multisystemic phenotypic effects? Numerous mechanisms have been proposed (Figure 1) including (i) aberrant alternative splicing of several key mRNAs (21–32), (ii) altered transcriptional regulation (30,33–36), (iii) repeat-associated non-ATG translational initiation on CUG-expansions producing poly(Q) peptides in DM1 cells (37), (iv) CUG-hairpin-induced stress pathways that inhibit translation in DM1 (4,38–40), (v) CUG-expansion-dependent dysregulation of miRNA processing/function (36,41,42) and finally (vi) alterations in the usage of alternative polyadenylation sites of a number of mRNAs (43). These modes of regulation are not mutually exclusive and may all contribute significantly to the pathogenesis of DM1. Central to all these mechanisms is the homeostasis of CUG-expanded DMPK mRNA, which, depending on the cell type, can be found in both the nucleus and the cytoplasm (44,45), where it, in complex with RNA-binding proteins, can form large and dynamic aggregates or foci (23,46–48). More specifically, in dividing DM1 fibroblasts, a larger fraction of the CUG-expanded transcripts reach the cytoplasm, where it can nucleate RNA-binding proteins (44,45,47) and trigger stress responses involving PKR-mediated phosphorylation of eIF2α, which in turn causes a global reduction in translation (4,38). Furthermore, recent evidence from using a *Caenorhabditis elegans* DM1 model suggests that a key nonsense-mediated mRNA decay (NMD) factor *smg-2* (Upf1 in *Homo sapiens*) can affect the homeostasis of CUG-expanded nuclear foci (49).

In this review, we focus on the dynamic regulation of CUG-expanded mRNA foci and how the CUG-associated RNA-binding proteins can influence and deregulate DM1-specific mRNA processing events.

### Misregulated mRNA processing and decay in DM1

A well-established consequence of CUG-expanded DMPK mRNA expression, and a paramount feature of DM1 pathology, appears to be aberrant splicing of numerous mRNAs, including the ones encoding the insulin receptor (IR2), the chloride channel (ClC2) and the cardiac troponin T (cTNNT2) (3). Several RNA-binding proteins including the muscleblind-like protein 1 (MBNL1) and the CUG-triplet repeat RNA-binding protein 1 (CUGBP1) have been recognized as important players in regulating DM1-specific aberrant splicing, consistent with their ability to interact with CUG-repeats (21–32). These known splicing regulators generally exert opposing effects onto many of their respective pre-mRNA splicing targets during development (50–53). Thus, in adult DM1 tissues inactivation of MBNL1 function by intranuclear sequestration in foci (53–56) and a concomitant increased stability of CUG-BP1 due to PKC-dependent phosphorylation in most affected tissues (24,28) drives the production of normally embryonically expressed splicevariants. A subset of recently identified MBNL1 interaction-partners, hnRNP H, H2, H3, F and DDX5, are all found to be upregulated in both DM1 myoblast and cells expressing CUG-expanded mRNA and appear to be important for splicing deregulation (31). Further evidence for severe MBNL1-dependent splicing defects in DM1 was found by globally comparing alternative splicing events in a MBNL1 knockout mouse model (57) to that of a model expressing CUG-expanded mRNA (16), both of which recapitulate central DM1 phenotypes. Specifically, these analyses, which were based on splicing-sensitive microarrays, indicated that >80% of the hundreds of splicing defects induced by CUG-expanded mRNA could be explained by loss-of-function of MBNL1 (30). In both mouse models, the CUG-BP1 levels are high relative to functional MBNL1, and this CUG-BP1/MBNL1 ratio has been proposed to be deciding for the degree of aberrancy of alternative splicing (1,11). In agreement with this notion is the finding that transgenic mice overexpressing CUG-BP1 also recapitulate classical DM1 features in various tissues (21,29,58). While much attention has been given to misregulated splicing events in DM1, CUG-BP1 and MBNL-proteins also interact with the 3′UTRs of numerous targets to tightly regulate both mRNA turnover rates and alternative polyadenylation (43,59–61). Aside from several important mRNA targets of relevance to DM1-specific phenotypes, an interesting auto-regulatory loop that impacts MBNL1/CUG-BP1 ratio was recently proposed based on the observation that MBNL1 and CUG-BP1 bind to each others 3′UTRs to promote mRNA decay of their respective targets (59). This ultimately may contribute to the well-documented upregulation of CUG-BP1 in most DM1 cells where most MBNL1 is sequestered, leading to stabilization of the CUG-BP1 mRNA. In addition, a recent study globally mapping alternative poly(A) sites in the absence of MBNL-proteins showed that these proteins differentially regulate alternative poly(A) site selection in hundreds of mRNAs via interactions upstream or in the vicinity of alternative poly(A) sites (43). Interestingly, these poly(A) sites are highly regulated during mouse embryogenesis and misregulated in DM1 muscle cells (43). It is evident that most 3′UTRs harbor regulatory elements important for proper mRNA localization, translation efficiency and turnover. Thus, the impact of sequestering MBNL1 in DM1 cells, thereby altering the intrinsic properties of numerous important mRNAs, whose products affect for example central protein translation/catabolism pathways, suggests that this phenomenon is potentially a major player in DM1 pathomechanisms (43).

### Ribonuclear foci

**Figure 2.**
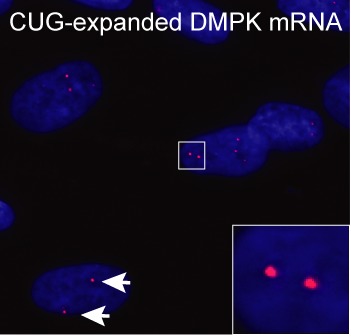
CUG-foci visualized by RNA-fluorescence *in-situ* hybridization. CUG-expanded DMPK mRNA accumulates in distinct foci in DM1 patient cells (red foci indicated at the tip of white arrows and enlarged in inset). Blue color indicates nucleus (DAPI staining).

A hallmark feature in DM1 is the accumulation of CUG-expanded mRNA in distinct nuclear aggregates, or foci, in various cells isolated from patients or cell lines expressing CUG-expansions (Figure 2) (23,44–48,54–56,62–67). In a pioneering study of DMPK mRNA localization in normal and DM1 skin fibroblasts Taneja *et al*. (45) demonstrated that the CUG-expanded DMPK mRNA transcribed from both the normal and mutated allele also resides in the perinuclear region of the cell cytoplasm. It is still unclear whether CUG-expanded mRNAs are actively, but inefficiently exported to the cytoplasm or whether they are merely leaking into the cytoplasm during nuclear envelope breakdown in dividing fibroblasts. Further characterization of the nuclear foci in non-dividing differentiated muscle cells revealed that the CUG-expanded DMPK mRNA is spliced/polyadenylated and yet virtually confined to the nucleus as judged by Northern blotting (44). Interestingly, mRNA decay assays using prolonged inhibition of transcription with Actinomycin D demonstrated a rather slow decay rate (*t*_1/2_ >13 h) for both the CUG-expanded and normal DMPK mRNA, which is consistent with the finding that nuclear foci could still be detected after 15 h of transcription inhibition (44). It is unclear why the CUG-expanded mRNAs are inefficiently exported from the nucleus of non-dividing muscle cells (44), but hnRNP H has been proposed to facilitate nuclear retention of CUG-expanded mRNPs, since hnRNP H knockdown enhances expression from an otherwise poorly expressed GFP reporter containing 85 CUG repeats in primary myoblasts (68). In line with this, hnRNP H had previously been shown to co-localize with CUG-expanded mRNA (69) and was able to regulate IR2 splicing in complex with CUGBP1 and MBNL1 (32). Thus, accumulation of the nuclear foci in the periphery of nuclear speckles along with a nuclear matrix association may hamper normal mRNA export pathways by unknown mechanisms (44,63). Moreover, evidence suggests that slow nuclear diffusion rates of CUG-expanded mRNPs may explain why the nuclear export is rather inefficient (48). Interestingly, a recent study has shown that the double-strand RNA-binding protein, Staufen 1, is upregulated in DM1 muscle tissue where it interacts with CUG-expansions, counteracts key DM1-specific splicing events and enhances the nuclear export and translation of GFP reporters containing 200 CUG repeats (70). Importantly, these activities were reported to be independent of CUG-foci homeostasis (70).

Early studies reported co-localization of CUG-foci with muscleblind family proteins (MBNL1, MBNL2 and MBNL3) (54,55,65), consistent with the ability of MBNL proteins to directly interact with the CUG-expansions (71) and recently reviewed in (72). These studies did not, however, address whether co-localization between MBNL1 protein and CUG-foci (or their direct interaction) is required for the adverse splicing events observed in DM1, *per se*. The finding that MBNL knockout mice and CUG-BP1 overexpressing transgenic mice can recapitulate most DM1 phenotypic features, in the absence of expanded CUG-repeats, and henceforth foci, suggests that foci *per se* are not required for DM1 pathogenesis (21,29,73,74). In line with this, expression of ‘foci-forming’ CAG-expanded mRNAs in COSM6 cells failed to misregulate splicing of cTNT or IR2 pre-mRNAs suggesting that foci formation and misregulation of splicing are separable events (23). By contrast, another study reported that expression of CAG-repeats functionally mimics CUG-mediated mis-splicing in HeLa or SK-N-MC cells (75). This discrepancy can likely be explained by differences in expression levels of reporters and endogenous splicing factors in the different cell lines. Taken together, these results indicate that foci formation is a consequence of CUG-expansion and one way of ‘inactivating’ MBNL1, but is in itself not a prerequisite for misregulation of splicing. Rather, the intricate balance between CUG-BP1 and functional MBNL1 seems to determine the degree of aberrancy of splicing in DM1, irrespective of whether this balance is skewed by nuclear foci or not (1,11,76). However, this does not change the well-documented correlation between foci formation in cells expressing CUG-expansions and their DM1-specific misregulation of splicing. The CUG-expanded mRNPs have been suggested to exist in both a diffuse (soluble) and a focus (insoluble) form, of which the latter primarily nucleates MBNL1, which in turn might exclude other CUG-mRNP-interacting proteins such as CUG-BP1 (11,28). Thus, the apparent lack of co-localization between foci and any RNA-binding protein potentially important in DM1 pathogenesis (e.g. CUG-BP1) may merely reflect their enhanced interaction with the soluble/diffuse form of the CUG-expanded mRNP (11). Such an appealing model argues for a dynamic transition between the focus and the diffuse form.

### Modulators of ribonuclear foci

A recent study used a reporter system, which enables kinetic assessments of GFP-labeled mRNPs in real time (MS2-coat protein fused to GFP binds to the mRNA), to elegantly demonstrate that CUG-expanded foci display a stochastic aggregation behavior where they assemble/disassemble at rather fast rates (within minutes), suggesting that these complexes are likely much more dynamic than first anticipated (48). Moreover, the ability to visualize single mRNPs revealed that CUG-expanded mRNPs (containing 145 CUGs) reside in both aggregated foci (200–1250 nm in size) and as single mRNPs (<200 nm in size), where both classes can adopt either a diffusive/corralled- or a static state (48). This finding lends support to the two-state model presented above (11). How are these mRNPs/foci then regulated? Aside from the MBNL proteins (and likely CUG-BP1) (11,48,62) DEAD-box helicases DDX5, DDX17 and DDX6, which are proteins with general mRNP remodeling capacity, have recently been shown to modulate CUG-expanded foci and may play a role in DM1 pathogenesis (46,47).

#### MBNL1/CUG-BP1

MBNL1 is probably the best-studied protein of the 3-member muscleblind like family of proteins (MBNL1-3). The protein is ubiquitously expressed (with the highest expression level in muscle tissue) and it contains four classical zinc-finger domains of the CCCH-type (reviewed in (72)). Evidence suggests that foci formation is MBNL1-dependent, since depletion of the protein is associated with decreased aggregation of CUG-expanded mRNA, consistent with its ability to interact with CUG-containing sequences, *in vitro* and *in vivo* (72). Several lines of evidence have identified a binding preference of MBNL proteins for a YGCY tetra-nucleotide (Y = pyrimidine), which confers high-affinity binding (30,38,40,59,61,72,77–79) and is well represented within the CUG-repeats in DM1. A recent crystal structure of two MBNL1 zinc-finger domains in complex with a short RNA oligomer (CGCUGU) demonstrates that the MBNL1 peptide primarily contacts the central GC dinucleotide (38), which, in the proposed CUG_n_ hairpin structure (12,15), is involved in Watson–Crick basepairing. This has led to the proposal that the short double-stranded regions within the CUG-expanded DMPK mRNA likely have to be unwound for MBNL1 to bind efficiently to this single-stranded region (38,72,80). Then how does MBNL1 interact strongly with CUG-repeats if the central GC is presumably basepaired? Another recent study suggested that MBNL1 prefers unpaired U-nucleotides, but efficiently binds RNAs with either unpaired or basepaired central GC-nucleotides in contrast to CUG-BP1, which prefers single-stranded RNAs (78). Consistent with this, although dismissed by many, due to the lack of co-localization between CUG-BP1 and the nuclear CUG-foci, it is very likely that CUG-BP1 in fact interacts with CUG-repeats in their single-stranded/soluble form, which in turn may contribute to the reported PKC-dependent upregulation in DM1 cells (11,24,28,81). Plausible models explaining how MBNL1 is able to interact with DMPK CUG-expansions and produce foci have been proposed (72). One of these models involves local unpairing/unwinding of the central GC-pairs contacted by one of the four MBNL1 zinc-fingers, which may allow bridging between multiple CUG-hairpins (72). A further stabilization of such complexes might be carried out by the ability of MBNL1 to dimerize or even self-aggregate (77,79). Since these models are primarily based on *in vitro* studies additional factors may influence MBNL1 (or CUG-BP1) binding and foci formation, including some of its recently identified interaction partners (31,46).

#### DDX5/DDX17

MBNL1 was recently reported to interact with several proteins in an RNA-independent manner: hnRNP H, H2, H3, F, A2/B1, K, L, DDX5, DDX17 and DHX9, which were all found to be upregulated in both DM1 myoblasts or cells expressing CUG-expanded mRNA (31). Interestingly, the DEAD-box family proteins DDX5 (p68) and DDX17 (p72) were also identified by mass spectrometry in complexes assembled onto *in vitro* transcribed CUG-repeats (46). Both exogenous and endogenous DDX5 and DDX17 were shown to co-localize with exogenous CUG-expanded reporters expressed in various cell types, but this could not be recapitulated when staining endogenous factors in DM1 myoblasts (46). The DEAD-box family of RNA helicases is characterized by encompassing a conserved core of nine distinct motifs, including the DEAD-box signature. These helicases are involved in numerous aspects of mRNA metabolism including transcription, nuclear export, translation and mRNA decay (reviewed in (82)). Homologous DEAD-box helicases have been reported to exert rather diverse functions, including stable RNA-binding (clamping) of larger complexes while others transiently interact with RNA and through ATP-hydrolysis unwind local RNA structures and in some cases displace proteins (82). Interestingly, using *in vitro* binding assays with purified components, Laurent *et al*. elegantly demonstrated that DDX5 strongly enhanced the interaction of MBNL1 with RNAs containing CUG-, CAG- or CCUG-repeats, and that this relies on an intact helicase-domain, suggesting that DDX5 is indeed a modifier of MNBL1 binding activity (46). It is plausible that DDX5 (and potentially DDX17, which was not tested) may locally unwind the central basepaired GC-dinucleotides, which in turn may increase the affinity of MBNL1 (38,46). Moreover, it was found that DDX5 was involved in regulating alternative splicing of TNNT2, but not of IR2 or ATP2A1, in an MBNL1-dependent manner. This suggests that DDX5 is involved in a subset of splicing events associated with the pathology of DM1 and further strengthens the hypothesis of MBNL1 having a central role in disease development. However, it also indicates that despite the interaction of DDX5/DDX17 with CUG-repeats and regulation of MBNL1 binding to these *in vitro*, DDX5 likely confers rather subtle modulatory effects on MBNL1 binding to CUG-repeats *in vivo*, where other factors and more stringent conditions might compete for the DDX5/DDX17 activities. However, knockdown of DDX5 modestly reduced foci formation when using an inducible reporter mRNA containing 960 CUG-repeats (46). Furthermore, the finding that endogenous DDX5 does not efficiently co-localize with CUG-foci in DM1 myoblasts (46) indicates that their interaction may be transient or that DDX5 interacts mainly with the soluble form of CUG-expanded mRNPs (11). Another possibility is that once MBNL1 becomes ‘delivered’ and bound to the UGCU-sequence DDX5 dissociates from a larger complex.

#### DDX6 and Upf1

**Figure 3.**
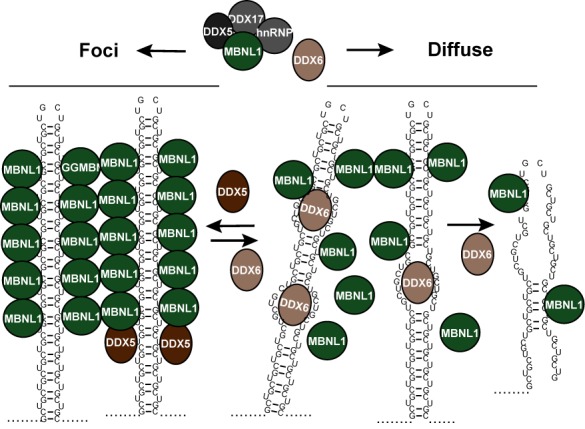
Two-state model of CUG-expansions in foci or diffuse states. DDX5 and DDX6 exert opposite effects on CUG-foci potentially by promoting (DDX5) or restricting (DDX6) MBNL1 binding to CUG-repeats allowing for dynamic changes between a focal/insoluble state and a diffuse/soluble state (see text for further details).

A recent study supports the notion of a more general function of DEAD-box helicases in DM1 by showing that DDX6 can modulate foci homeostasis in DM1 cells (47). Specifically, knockdown of DDX6 increased the frequency and intensity of CUG-foci in DM1 fibroblasts, which was not a result of a change in the MBNL1 or the CUG-expanded DMPK levels. Conversely, overexpression of DDX6 reduced foci count and increased the occurrence of cytoplasmic RNA-signal when assessed by RNA-FISH (47). Interestingly, fibroblast isolated from DM1 patients with an excess of 2000 CUG-repeats also showed rare but distinct cytoplasmic foci (47) as reported previously by others using exogenous CUG-reporters (47,83,84). These foci did not coincide with well-known cytoplasmic RNA-granules called processing bodies (47), which harbor key mRNA decay factors such as decapping enzymes and 5′–>3′ exonucleases (85–87). Although only a very small fraction of the cellular pool of DDX6 could be detected in the periphery of nuclear foci, CUG-expanded DMPK mRNA was highly enriched over wild-type DMPK mRNA, when performing DDX6 immunoprecipitations followed by qRT-PCR, consistent with the ability of DDX6 to directly interact with CUG RNAs *in vitro* (47). These results indicate that DDX6 may counteract the effects of DDX5 (Figure 3). In line with this, a partial relief of misregulated splicing of the IR2 pre-mRNA was observed upon DDX6 overexpression (47). Further studies, using global approaches, will likely decide whether this effect can be extrapolated to more mis-splicing events observed in DM1 (30). DDX6, purified from large-scale cultures of stable HEK293 cells, can unwind CUG-repeats *in vitro* suggesting that DDX6 may modulate (i.e. diminish) MBNL1 association with CUG-expanded mRNAs. Importantly, the displacement of two pre-annealed CUG-strands required ATP and an intact DEAD-box motif. Taken together with the finding that DDX6 overexpression leads to a more diffuse staining of MBNL1 in the majority of DM1 cells, it was concluded that DDX6, although being a mainly cytoplasmic protein, is able to locally unwind CUG-hairpin structures in the nucleus and potentially displace MBNL1 from the repeats (47). Future studies will decide whether the DDX6-mediated occurrence of more cytoplasmic CUG-expanded RNA in the cytoplasm reflects a transition from focus-to-diffuse states of CUG-mRNPs (11), potentially allowing for increased binding to CUG-BP1 and/or export to the cytoplasm. In line with such a scenario, Staufen 1 was recently shown to bind and increase nuclear export and translation of GFP reporters containing 200 CUG repeats upon overexpression (70). However, once the CUG-expanded DMPK mRNA has been exported to the cytoplasm, little is known about its translation efficiency and decay rates. The initially reported low cytoplasmic levels of CUG-expanded DMPK mRNA (44) could potentially be caused by fast cytoplasmic decay rates, which is consistent with the ability of both CUG-BP1 and MBNL1 to promote mRNA decay of their targets (59,88–90). Supporting such a scenario, inclusion of CUG-repeats in a GFP reporter has been shown to lower steady-state GFP-mRNA levels approximately 5-fold compared to a non-expanded reporter, which suggests that CUG-repeats enhance decay rates (70). One possibility is that the partially double-stranded CUG-repeat serves as a binding platform for Staufen 1 (70), which will not only enhance mRNA nuclear export and cytoplasmic translation, but eventually also promote mRNA decay by inducing Staufen-mediated decay (SMD) (91). SMD is a prominent process in myoblasts and involves Staufen-mediated recruitment of a large RNA helicase called Upf1, which is known as an essential factor for the cytoplasmic surveillance and clearance of mRNAs containing premature termination codons by the process of NMD (91,92). Further evidence for a function of the NMD/SMD factor Upf1 in CUG-foci dynamics has come from a study using RNAi libraries to screen for modifiers of CUG-foci in a *C. elegans* DM1 model (49). Specifically, knockdown of the *C. elegans* orthologue of Upf1, *smg-2*, was shown to significantly increase both the levels of a model CUG-expanded transcript and cytoplasmic as well as nuclear CUG-foci frequency in the worm muscle cells (49). A similar phenotype with increased frequency of nuclear CUG-foci was obtained upon knockdown of Upf1 in human DM1 myoblasts (49). Whether these effects are caused by direct stabilization of CUG-expanded transcripts in the cytoplasm upon inhibition of SMD and NMD (i.e. knockdown of *smg-2*/Upf1) or whether these are caused indirectly through other NMD/SMD regulated factors remains to be fully clarified.

## CONCLUSION AND FUTURE PERSPECTIVES

Although DM1 is among the well-characterized muscle dystrophies, many questions remain unanswered and some of the defining molecular features of the disease are continuously being debated. For example, although the distinct foci in the nucleus are considered one of the key molecular hallmarks of the disease, their role in DM1 pathology is not well understood. Are these RNA aggregates necessary for MBNL1 sequestration or rather a consequence of MBNL1 binding and aggregation? It is evident that there is a strong correlation between foci frequency and the level of functional MBNL1 impairment, although inactivation of MBNL1 in the absence of foci (i.e. knockdown or in knockout mice) produces key features of DM1. In addition, the fact that CUG-BP1 overexpression produces a similar phenotype argues for a mechanism in which the ratio between functional MBNL1 and CUG-BP1 primarily initiates pathogenic features. The dynamic assembly/disassembly of CUG-foci, which can take place within minutes, and the implication of DEAD-box helicases in their homeostasis, corroborates a model that CUG-expanded mRNPs exist in either a soluble or an insoluble state. The general solubility of CUG-expanded mRNPs may also regulate the efficiency of their nuclear export, translation efficiency and decay rate. Further investigations of the impact of enhanced cytoplasmic CUG-expanded mRNA turnover via SMD/NMD and the potential dependence on DEAD-box helicases will therefore be important future directions. Future studies will likely also reveal additional modulators of CUG-foci in DM1 and decide whether these impact RNA binding of MBNL1 or CUG-BP1 and thereby the aberrant gene expression observed in DM1.
